# Technology Adoption Among Seniors During COVID-19 Pandemic Impacts Mental Health and Feelings of Companionship

**DOI:** 10.1093/geroni/igaa057.3526

**Published:** 2020-12-16

**Authors:** Brittany Derynda, Mary Goodyear, Jade Kushner, Nicole Cook

**Affiliations:** 1 Nova Southeastern University Dr. Kiran C Patel College of Osteopathic Medicine, Clearwater, Florida, United States; 2 Nova Southeastern University Dr. Kiran C Patel College of Osteopathic Medicine, Fort Lauderdale, Florida, United States; 3 College of Psychology, Fort Lauderdale, Florida, United States; 4 Nova Southeastern University, Davie, Florida, United States

## Abstract

Social isolation and lack of companionship, exacerbated by COVID-19 “stay at home” orders, has been an ongoing concern among seniors in the US. Among other strategies, Lifelong Learning Institutes (LLIs) were created to support continuing education for older adults. These programs bring seniors together to encourage engagement through lectures, art and fitness classes in a common space. LLI in South Florida adapted to COVID-19 “stay at home” orders by moving all programming online in March 2020. In May 2020 LLI members, faculty and students designed a research study to understand the experience of LLI members with social isolation and companionship prior to, and during, “stay at home” orders. Responses included 127 members (mean age 75.5). Respondents reported significantly lower social isolation (p<.01) and lack of companionship (p<.01) as a result of “stay at home”. Interestingly, social isolation had no significant explanatory variables. However, significant results (p<.05) showed that seniors who isolated alone were 6.7 times more likely to lack companionship compared to those who isolated with a friend or spouse; seniors who reported they are not tech savvy were 8.3 times more likely to lack companionship compared to those who reported they are tech savvy; and that for every additional day of poor mental health respondents had a 1.15 higher odds of lacking companionship. These results underscore the importance of technology adoption among seniors during times of social isolation and the positive impact this can have on companionship and mental health.

